# A Low-Power WSN Protocol with ADR and TP Hybrid Control

**DOI:** 10.3390/s20205767

**Published:** 2020-10-12

**Authors:** Chung-Wen Hung, Hao-Jun Zhang, Wen-Ting Hsu, Yi-Da Zhuang

**Affiliations:** Department of Electrical Engineering, National Yunlin University of Science and Technology, 123 University Road, Section 3, Douliou, Yunlin 64002, Taiwan; m10512027@yuntech.edu.tw (H.-J.Z.); m10712051@yuntech.edu.tw (W.-T.H.); b10512118@yuntech.edu.tw (Y.-D.Z.)

**Keywords:** adaptive data rate (ADR), transmit power control (TPC), time division multiple access (TDMA), wireless sensor network (WSN), power consumption, Internet of Things (IoT)

## Abstract

Most Internet of Things (IoT) systems are based on the wireless sensor network (WSN) due to the reduction of the cable layout cost. However, the battery life of nodes is a key issue when the node is powered by a battery. A Low-Power WSN Protocol with ADR and TP Hybrid Control is proposed in this paper to improve battery life significantly. Besides, techniques including the Sub-1GHz star topology network with Time Division Multiple Access (TDMA), adaptive data rate (ADR), and transmission power control (TPC) are also used. The long-term testing results show that the nodes with the proposed algorithm can balance the communication quality and low power consumption simultaneously. The experimental results also show that the power consumption of the node with the algorithm was reduced by 38.46-54.44% compared with the control group. If using AAA battery with 1200 mAh, the node could run approximately 4.2 years with the proposed hybrid control algorithm with an acquisition period of under 5 s.

## 1. Introduction

The wireless sensor network (WSN) is the one of bases in Internet of Things (IoT), and most nodes in the WSN are powered by battery. Extending battery life, saving the maintenance fee, and raising system reliability are the motivations of this paper. In addition to the battery technology improvement and power capacity increase, the low-power technology of the device is also significant. In many applications, IoT device’s security and power consumption are significant issues [1]. The battery power is usually used in the application of IoT devices, and the battery life is a troublesome problem [2]. Therefore, achieving extremely low power consumption on battery IoT devices has also become a big challenge.

Moreover, wireless transmission is the highest power-consuming process in communication devices. A study pointed out that the power consumption of nodes in the wireless sensing network is mostly concentrated in the process of wireless communication [3]. Therefore, maintaining reliable communication quality and reducing the power consumption of the device through wireless communication optimization is the focus of this paper. A WSN structure-integrated time division multiple access (TDMA), transmission power control (TPC), and adaptive data rate (ADR) are proposed in this paper to reduce the power consumption of wireless communication.

This paper is based on previously published research by this paper’s authors, which has discussed the relationship and performance analysis of the transmission power and data rate [4]. This paper then implements an ultra-low-power WSN IoT with transmission power and data rate hybrid control and introduces the details. The predecessor of this paper aimed to evaluate the performance difference between the transmission power and the data rate with the same packet error rate (PER) of 1% through sensitivity measurement, thereby achieving a comparison basis of hybrid control between data rate and transmission power [4]. In this paper, TDMA scheduling detail, data rate parameter settings, low current selection of transmission power, a simplified hybrid control algorithm, and practical application are discussed. Finally, this paper also places multiple sensing nodes and measures the energy-saving effect and PER state of the nodes, and the proposed hybrid control algorithm is expected to achieve reliable wireless communication and extremely low power consumption simultaneously.

In summary, this paper provides the following contributions:

1. We propose a hybrid control algorithm combined with TPC and ADR that could adapt the environmental interferences.
2. Experimental results analysis show that the proposed algorithm achieved energy-saving with stable communication quality.

## 2. Related Research

The architecture of the WSN in IoT application and the selection of communication frequency bands have been discussed in the following studies. The designs for power consumption reduction in the wireless network, such as media access control (MAC), transmission power, and data rate control, have also been also discussed in the following literature.

There are two data processing methods that have been proposed, centralization and distribution data fusion, which each have different benefits. Centralized data fusion processes all the data on a central node, while the nodes in distributed system process their own data [5]. In order to keep high maintainability and easy data processing, we adapted star topology network in this paper.

Compared with the 2.4 GHz or 5 GHz frequency band, the transmission distance of the Sub-1 GHz wireless communication is farther, so its coverage is wider and its power consumption is lower [6,7,8]. The method of multinodes communication in the WSN includes competition-based [9,10,11,12] and scheduling-based MAC [13,14,15,16]. Compared with competition-based MAC, scheduling-based MAC network throughput is not good, but the design of scheduling-based MAC is simpler. The authors of [17] proposed a TDMA structure-based MAC protocol for short and long-range networks, and the sensor node can run for about 3 years. TDMA could reduce dramatic the power consumption of the WSN, as shown in [17]. However, the proposed algorithm in this paper including ADR and TP control would lower the power requirement further. Considering the system complexity and low-power design, TDMA was adopted in this paper.

In wireless communication, transmission power is a major factor for power consumption. A method called TPC minimizes the transmission power as possible when the communication quality can be maintained [16,17,18,19,20,21,22,23,24]. If the environment is better, TPC can achieve more power-saving effects. In addition to the transmission power, the data rate is also a major factor affecting power consumption. In the case of packet transmission, if the transmission data rate is faster, the wireless transmission time is shorter. However, the cost of a faster data rate is transmission quality reduction. The appropriate data rate is selected based on the relative relationship between the frame delivery ratio (FDR) and the received signal strength indicator (RSSI) [25,26,27]. Sodhro, A.H. et al., [28] proposed an energy-efficient transmission power control (ETPC) algorithm that was based on a wireless channel estimation. The channel estimation is an important issue for communication quality and power consumption. The results of the wearable electrocardiogram, demonstrated in [28], have been validated successfully and make a great contribution in energy-saving and signal-processing. However, the complex control should be considered. The methods of combining data rate and transmission power control to achieve power saving were proposed in [29,30]. Due to the fact that ADR and TP hybrid control is complex, and there are a lack of lectures discussing it, there are research gaps to be covered in this paper. The authors of [31] also considered DSSS and MFSK for communication quality and power consumption. However, the proposed algorithm was based on the decided communication to select a suitable combination of DSSS and MFSK. In order to adapt the environmental interference, a data rate and transmission power-integrated control algorithm was proposed in this paper. The control algorithm can adapt to the environment in which the sensor node is located and choose the best data rate and transmission power. However, the previous works have discussed the TP and ADR control individually, but there is a lack of discussion on combination control due to its complexity. Moreover, because ADR control under TDMA is more difficult because of the complicated communication handshaking, few researchers have discussed the topic. In this paper, the TP or ADR combination control TDMA are detailed. 

## 3. System Implementation

### 3.1. System Architeture

**Figure 1:**
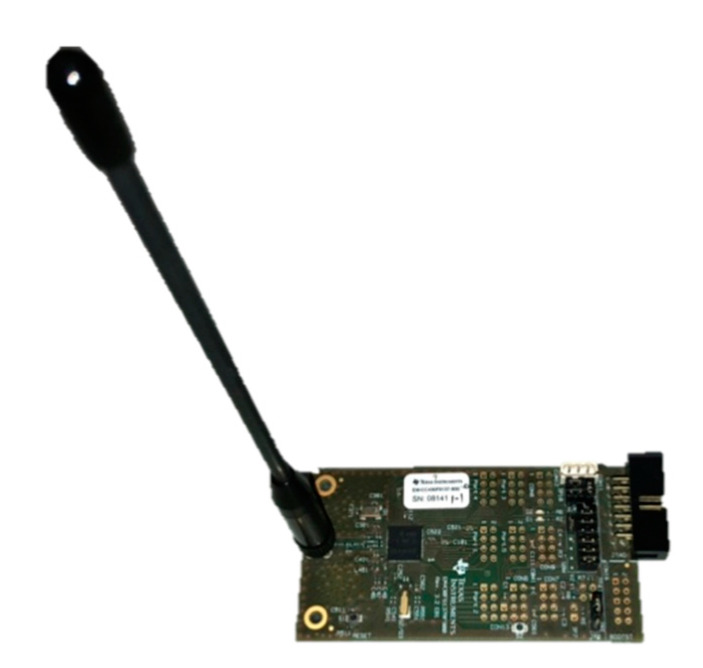
CC430F6137 development board.

**Figure 2:**
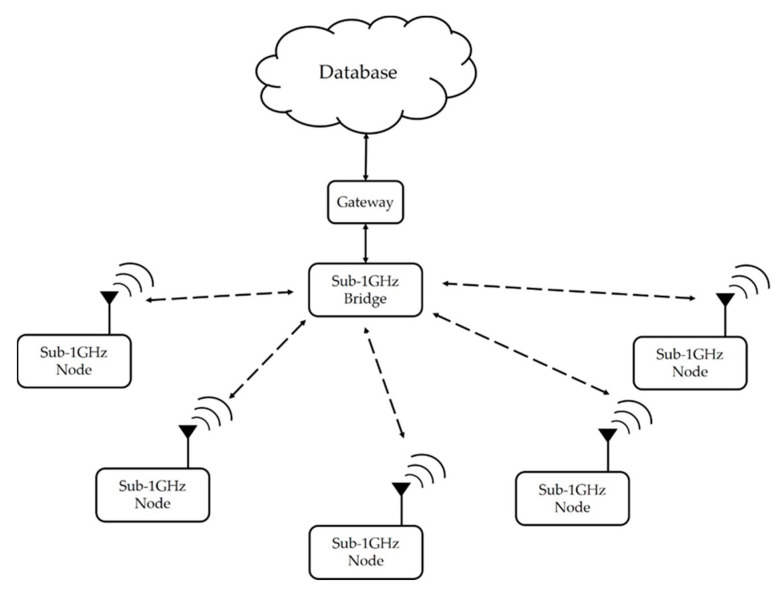
Star network architecture.

**Figure 3:**
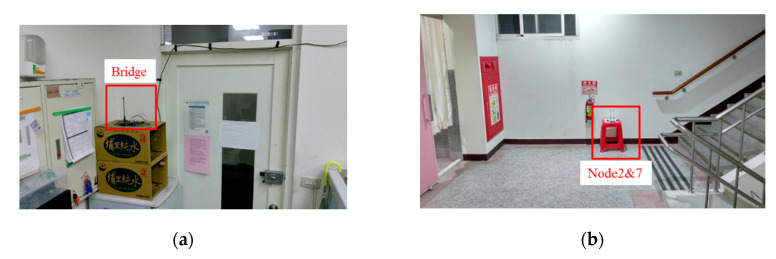
(**a**) The placement of the bridge; (**b**) the placement of node 2 and node 7.

Figure 1 is the implementation platform of Texas Instruments CC430F6137. The network architecture is a simple star scheme, as shown in Figure 2, and the frequency-shift keying (FSK)/Gaussian frequency-shift keying (GFSK) was selected as the radio modulation method. The network consisted of multiple sensing nodes and a central bridge. The bridge was supplied by grid power and the sensor nodes were supplied by the battery. The placements of the bridge and nodes are shown in Figure 3. The bridge received packet from the nodes, and it was connected by cable to a gateway. The exact position is shown in Section 6.1. The TDMA protocol was proposed in this system, and the data rate and transmission power control algorithms were used to reduce the power consumption of the node to extend the battery life.

### 3.2. TDMA Protocol

**Figure 4:**
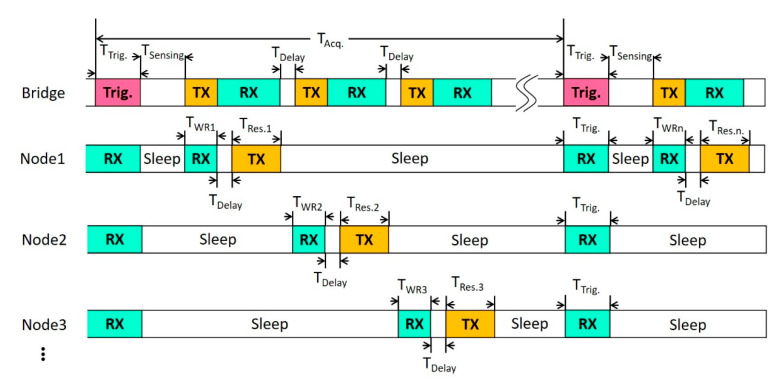
Diagram of TDMA architecture.

Figure 4 is a TDMA diagram proposed in this paper. The concept is to predetermine the communication time slot and turn off the wireless communication function when nodes are idle. The nodes enter the sleep mode for the rest of the time to save power. First, Trigger Time (TTrig.) is the synchronous trigger signal sent by the bridge. Next, Sensing Time (Tsensing) is the time interruption required by the node for sensing, and the time length depends on the processing time required by the installed sensors. Waiting for Require Time (TWRn) is the interval required by the nth node to wait for a bridge command. Response Time (TRes.n) is required to reply to the sensing information packet. Moreover, Delay Time (TDelay) is the slot for packet parsing, wireless communication reception, transmission mode switching, and radio wave calibration for the nodes and bridges. Finally, Acquisition Time (TAcq.) indicates the period from the start of the triggering to the end of the node polling.

The node ID is assigned before the network constructed, and the sequence of the TDMA time slot is dependent on this ID. When a connected sensor node is turned off, the bridge skips the slot after a couple reconnections. The reservation slot is reserved in the last portion of acquisition duration, and it is reserved for the connection of a new sensor node with lowest data rate and highest power. If the node is checked by the bridge, the time slot will be arranged into the dedicated ID slot in acquisition duration.

## 4. Measurement and Analysis of Radio Frequency

### 4.1. Parameters of Data Rate

The crystal oscillator used in this paper had a crystal oscillator error of 10 ppm. Texas Instruments provided the SmartRF Studio tool, which can set and select the appropriate frequency deviation and the receiving channel bandwidth at a specific data rate. The node update period of 5 s was used in this paper, the design goal was 255 nodes. Therefore, the slowest data rate could only be set to 26 kbps. In view of the above factors, the data rate of this paper was divided into nine segments, from fastest (250 kHz) to slowest (50 kHz), to obtain the parameters in Table 1. The paper adopted this method to set the required data rate and the correlation coefficient.

### 4.2. Receiver Sensitivity and Transmission Time in Different Data Rate

**Figure 5:**
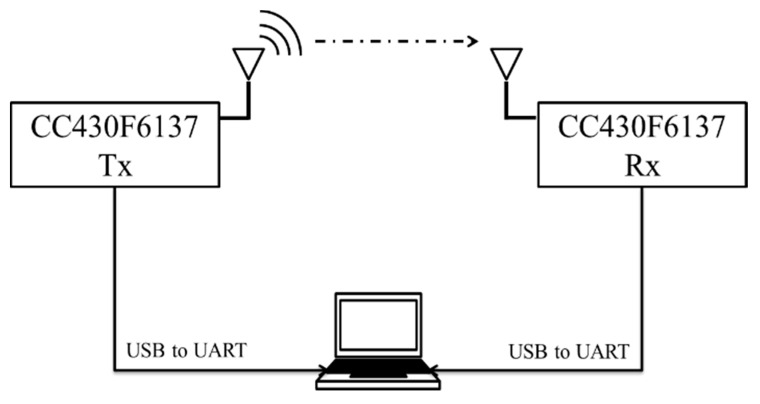
Experimental architecture of receiver sensitivity.

**Figure 6:**
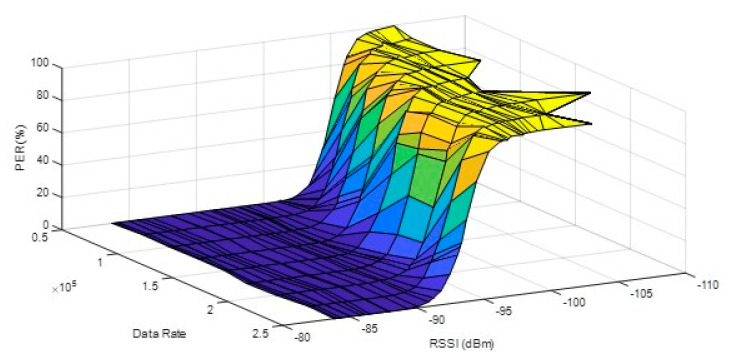
The relation between RSSI, data rate, and PER.

A sensitivity experiment was proposed in this paper, and the detail is shown in Figure 5. Two CC430 RF devices were used in this experiment: The transmitter and the receiver. On the transmitter, adjusting the transmission power is used to change the RSSI, 1000 packets are sent in a fixed data rate, and then average RSSI and PER are calculated on the receiver. In this paper, the corresponding RSSI when the PER was 1% was called sensitivity, and each data rate had a sensitivity. The relationship between data rate, RSSI, and PER is shown in Figure 6. The RSSI closest to one percent PER at each data rate was taken as the receiver sensitivity of the data rate, as shown in Table 2. The above Figure 6 relationship and Table 2 sensitivity table were used to analyze the wireless performance of different data rates.

### 4.3. Current Consumption in Different Transmission Power

The transmission powers of 121 segments are provided by the CC430F6137, Sub-1GHz wireless communication chip, while only 41 segments were selected in this paper. Due to the transmission power table provided by the original manufacturer, the actual value did not correspond to the 920 MHz band used in this paper. Therefore, the 121-segment transmission power values were measured at 920 MHz by the Rohde & Schwarz RTO2044 digital oscilloscope with a bandwidth of 4 GHz and a sampling rate of 20 GSa/s, and the more suitable 41 segment settings were chosen in this paper.

**Figure 7:**
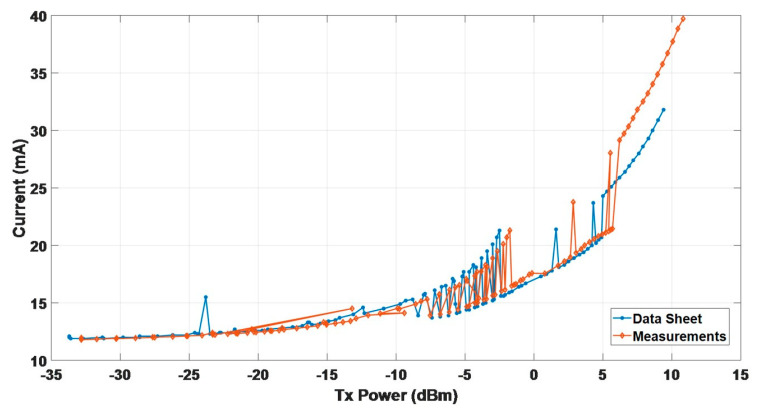
Comparison of the datasheet and measured values in the 121 segment transmission power.

Figure 7 is a chart comparing the measurement results of the current consumption corresponding to each transmission power with the original manufacturer. When the transmission power was larger, the difference between the values of the datasheet and the measurements was larger. In the interval where the transmit power was −9 dBm to 6 dBm, regardless of the values of datasheet or the measurements, the consumption variation was suddenly increased. Therefore, the appropriate 41 segment settings from the set value of 121 were selected, as shown in Table 3.

### 4.4. Total Power Consumption of Data Rate and Transmission Power

**Figure 8:**
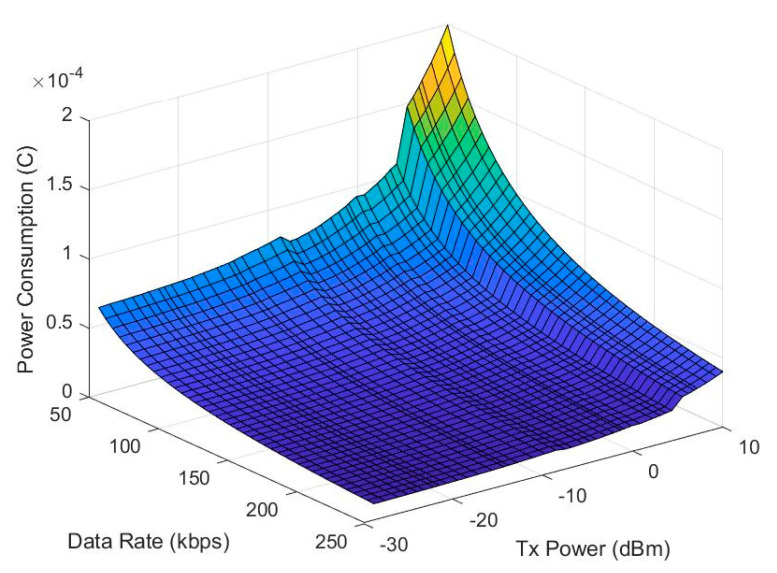
Relation of data rate, transmission power, and power consumption.

In Equation (1), I(TxPower) is the current consumption of the transmit power, and the relationship between the transmit power, data rate, and power consumption is shown in Figure 8 on the condition that the packet length is 33 Bytes. Figure 8 results were used for the method of energy efficiency comparisons in Section 5.

(1) $$Power\;Consumption=I\left(TxPower\right)\times Bit\;\mathrm{number}\times \frac{1}{Data\;Rate}$$

## 5. Control Algorithm

### Algorithm of Transmission Power and Data Rate Hybrid Control

**Figure 9:**
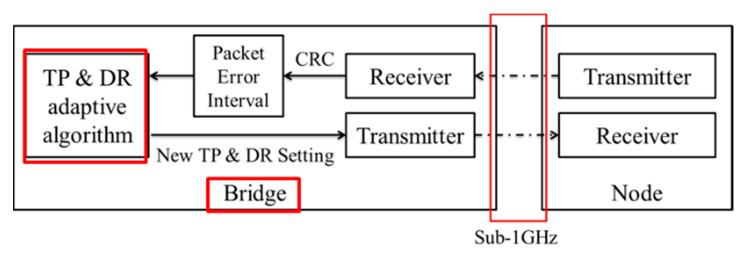
Control architecture between the bridge and sensor nodes.

**Figure 10:**
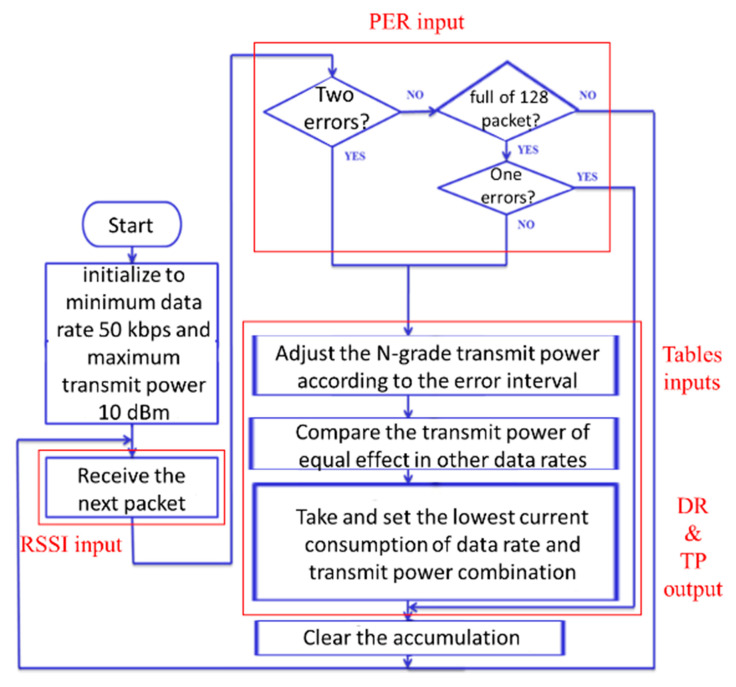
Flowchart of error interval algorithm controlling data rate and transmission power.

**Figure 11:**
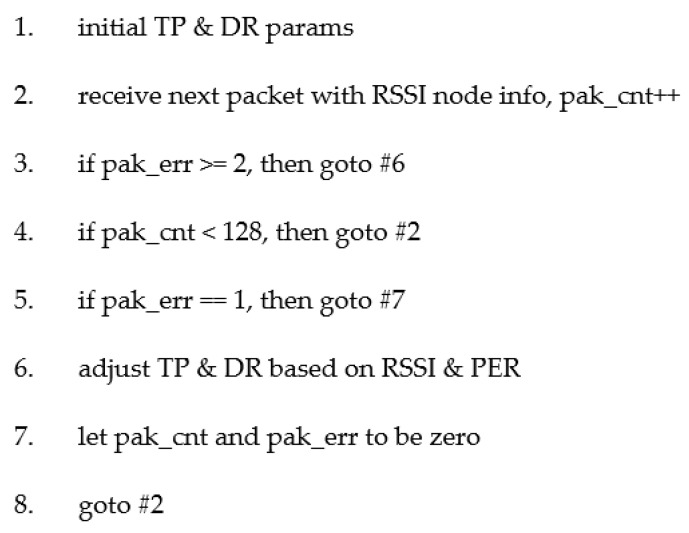
The pseudocode of control algorithm.

The transmission power and data rate hybrid control algorithm was proposed in this paper, and this algorithm was used to balance wireless quality and low power consumption to achieve a PER less than 1% and more power-saving. The control architecture between the bridge and sensor nodes is shown in Figure 9, and the hybrid control algorithm was run in Bridge. In Figure 9, the bridge received the RSSI feedback from sensor nodes’ transmission signals and calculated the PER using the packet error interval algorithm. After the hybrid control algorithm was complete, the new transmission power and data rate control command that generated by adaptive algorithm was sent to the sensor nodes from the bridge. The detailed flow of the hybrid control algorithm is shown in Figure 10 and its pseudocode is shown in Figure 11. In this system, input includes the real-time RSSI feedback, PER record, sensitivity table of different data rates, and power consumption table, and output includes the data rate control and transmission power control.

The communication quality target of this paper was set at a PER below 1%. In the algorithm of the packet error interval, the threshold of data rate and transmission power is adjusted by 128 packet durations. If there are no errors in the continuous 128 packets, it means that the PER is less than 1%. The data rate will be increased, or the transmission power will be reduced. However, if there is only one incorrect packet in a 128-packet period, it means that the PER is 1%, and the data rate and transmission power will not be changed.

Then, if two errors have occurred before 128 packets have been completed, the data rate will be reduced or the transmission power will be increased. In this algorithm, when the error interval is short, the larger amplitude of the transmission power is set, because it is necessary to react immediately when an error occurs. Otherwise, when the error interval is long, the lower amplitude of the transmission power is adjusted, because the environment may be stabilized and the error does not occur easily.

**Figure 12:**
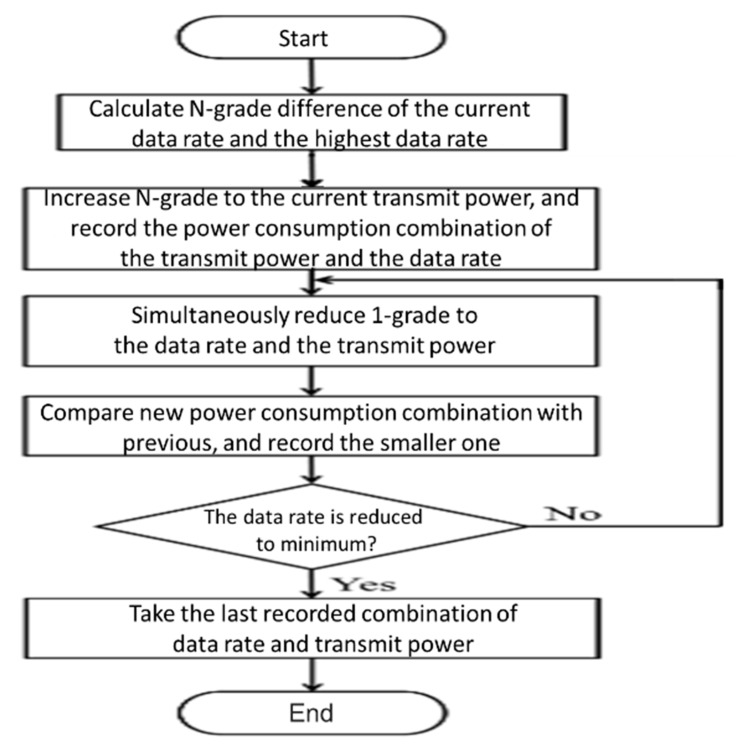
Flowchart for selecting the best energy efficient combination of data rate and transmit power.

Finally, by the error interval method, we determined how much to set the N-grade of data rate and transmission power for the next transmission. After the above N-order adjustment, based on the database of Figure 6 and Figure 8, the lowest power consumption combination of data rate and transmission power was selected as the result of the final adjustment. The method flow is shown in Figure 12.

## 6. Result

### 6.1. Experimental Method

**Figure 13:**
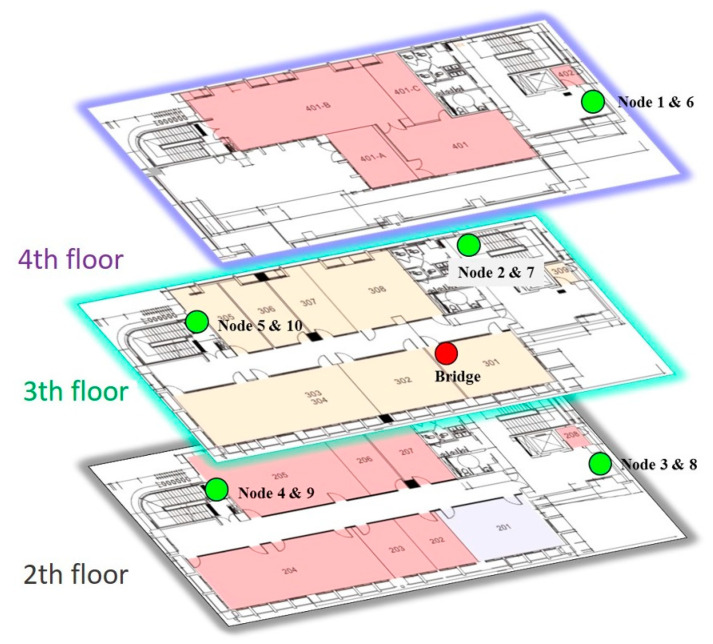
Experimental bridge and nodes placement map.

The experimental location of this paper is the Sixth Hall of Engineering, National Yunlin University of Science and Technology, Taiwan. A total of one bridge and ten nodes were set up in the experiment, as Figure 13 shows.

Every two nodes were placed in the same position. One node had the ADR and TPC control algorithms, and the other node fixed the data rate to the lowest (50 kbps) and the transmission power to the maximum. Through long-term testing, the power consumption and PER of nodes placed at the same position were compared to verify the effect of the algorithm. Nodes 1–5 were the nodes that had the algorithms, and nodes 6–10 comprised the experimental control group. A total of ten nodes were located in five different locations. Note that all nodes ran in the TDMA mode to save a lot of power for the sensing node. However, the power consumption of TDMA is not discussed later.

### 6.2. Results and Analysis

The experimental data of all nodes is organized as shown in Table 4. Since nodes 6 to 10 were without algorithms, the transmission power was set to a maximum of 10 dBm and the data rate was set to the lowest at 50 kbps. The PERs of nodes 6 to 10 were lower than the PERs of nodes 1 to 5 under the data rate and the transmission power control algorithm. However, the nodes with algorithms maintained a PER of less than 1% except for node 1, and the average current consumption was much lower than that the nodes without algorithms.

Comparing node 1 with node 6, the PER of node 1 was higher than node 6. However, node 1 saved 78.74% more power consumption than node 6 in response packet. In the overall average current consumption, node 1 saved 51.64% more of the energy than node 6.

Node 2 and node 7 were the nearest nodes for the bridge node. In Table 4, the PER of node 2 was 0.6322%, and node 7 was 0.1518%. Although the PER of node 2 was larger than that of node 7, its overall PER was still less than 1%. In the response packet, node 2 saved 73.67% more power consumption than node 7, and in the overall average current consumption, it saved 48.43% more power consumption.

The PER of node 3 was 0.9532%, and node 8 was 0.0912%. In the response packet, node 3 saved 70.49% more power consumption than node 8, and in the overall average current consumption, it saved 46.36% more power consumption.

**Figure 14:**
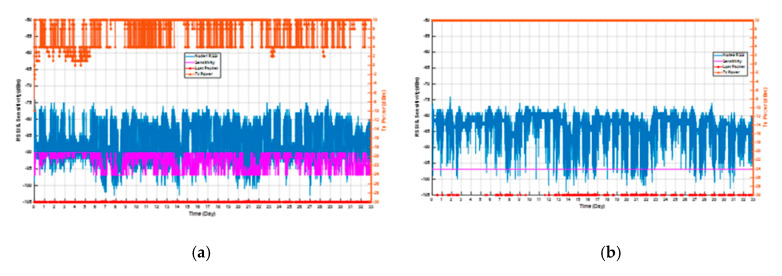
Experimental results of (**a**) node 4 and (**b**) node 9.

The experimental result is showed in Figure 14. The PER of node 4 was 0.8429%, and node 9 was 0.3331%. In the overall average current consumption, node 4 was 44.991 uA, and node 9 was 74.281 uA. In the response packet, node 4 saved 59.59% more power consumption than node 9, and in the overall average current consumption, it saved 39.43% more power consumption.

The PER of node 5 was 0.4011%, and node 10 was 0.3376%. In the overall average current consumption, node 5 was 32.516 uA, and node 10 was 74.281 uA. In the response packet, node 5 saved 85.83% more power consumption than node 10, and in the overall average current consumption, it saved 56.23% more power consumption.

Obviously, the RSSI values of nodes with algorithms are close to the sensitivity values corresponding to the current data rate from the experimental results. If the RSSI value is lower than the sensitivity value, the probability of packet error will increase. Moreover, since the position of the nodes is affected by the people in the office and the class, the RSSI value of each node floats dramatically during the daytime and the probability of packet error is high. However, at night, the RSSI value is so stable that the probability of packet error is low. If the node is supplied by AAA battery with 1200 mAh, the execution duration could approach about 4.21 years with the proposed algorithm. The nodes without the proposed algorithm could run only for 1.8 years.

**Figure 15:**
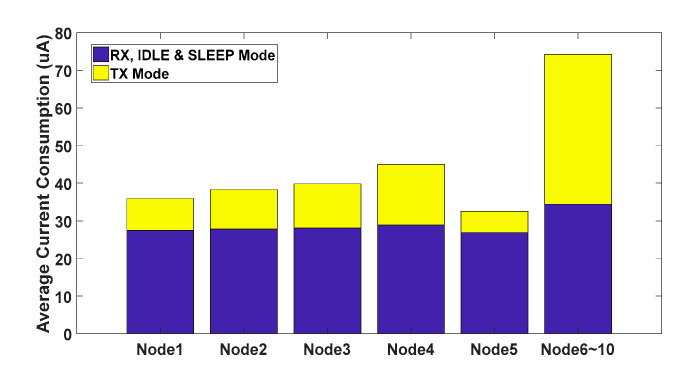
Nodes overall average current consumption.

The data of Table 4 is drawn with a bar graph of the nodes’ average current consumption as Figure 15. It is clear the response packet of the nodes with the algorithm saved the most significant energy in the TX Mode, and the ranking of the power saving was sequentially ranked as nodes 5, 1, 2, 3, and 4. The reason for the difference in the amount of power consumed by each node was assumed to be the positional relationship.

**Figure 16:**
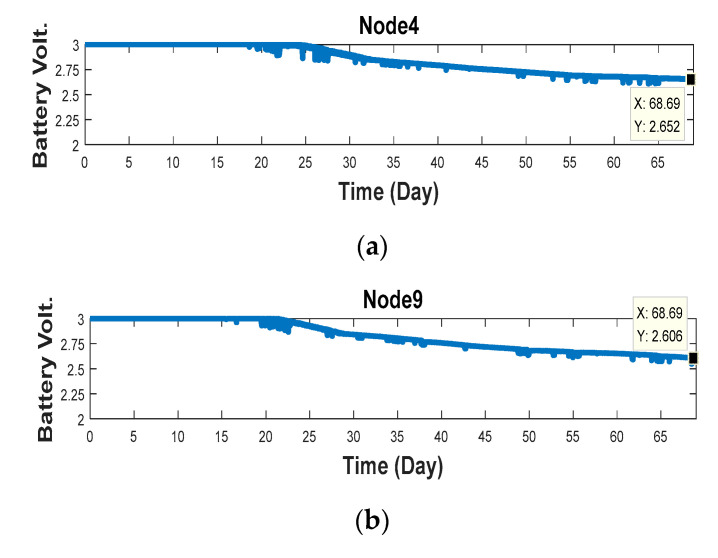
The battery voltage of (**a**) node 4 and (**b**) node 9 within 69 days.

For justification of the proposed algorithm, the experimental results for long-term testing are presented in Figure 16 and Table 5. Figure 16 shows the battery voltage variation in node 4 and node 9, which ran with and without control algorithm for 69 days of execution. The battery voltage of the node 9 in Table 5 was obviously lower than that of node 4, and the results also verify the proposed algorithm workable.

## 7. Conclusions

A wireless sensor network based on Sub1G-Hz and star topology was constructed in this paper, and the TDMA wireless communication protocol and the transmission power and data rate control algorithm were proposed to reduce power consumption on sensing nodes usefully. According to the dynamic environment, sensing nodes with hybrid control algorithms automatically adapt the transmission power and data rate to achieve good communication quality and low power consumption simultaneously. The above algorithms will increase the performance and reduce power consumption on wireless communication. Then, communication devices could be operated at very low power consumption when using wireless communication.

The experimental results show that PER states of nodes can effectively be controlled near the target value, 1%, which can prove the good reliability of communication. In addition, because all nodes run in the TDMA architecture’s wireless protocol, TDMA can enable a wireless transmission in a low duty cycle. The average current consumption of the node without the hybrid control algorithm was calculated as 74.281 uA, and the power consumptions of algorithm nodes were different and depended on the positions of the nodes. According to the experimental results, when the power consumption of the response packet in the transmission mode was compared, the power consumption saved up to 85.83%. The overall consumption saved up to 56.23% of the power consumption, which indicates that the algorithms proposed in this paper actually have an energy-saving effect for wireless communication. If the node is powered by AAA battery with 1200 mAh, the node could run approximately 4.21 years with proposed algorithm. The other TDMA is discussed in [17] for the LoRa system but not included about ADR and TP, and the authors suggest that the battery life is about 3 years.

In summary, the proposed hybrid control algorithm is complex, and the payload and node number in the WSN are also limited. However, the system architecture and control algorithm proposed in this paper could lower several important things such as the power consumption, system complexity, maintenance fee, etc.

## Figures and Tables

**Table 1:** Parameters of different data rates.

Data Rate	Frequency Deviation	RX BW
250 kbps	126.953125 kHz	541.666667 kHz
225 kbps	114.257812 kHz	464.285714 kHz
200 kbps	101.562500 kHz	406.250000 kHz
175 kbps	88.867188 kHz	406.250000 kHz
150 kbps	76.171875 kHz	325.000000 kHz
125 kbps	63.476562 kHz	270.833333 kHz
100 kbps	50.781250 kHz	232.142857 kHz
75 kbps	38.085938 kHz	162.500000 kHz
50 kbps	25.390625 kHz	116.071429 kHz

**Table 2:** RSSI when the PER was 1% at different data rates.

Data Rate	Sensitivity
50 kbps	−96.93 dBm
75 kbps	−95.22 dBm
100 kbps	−94.36 dBm
125 kbps	−93.69 dBm
150 kbps	−93.12 dBm
175 kbps	−91.96 dBm
200 kbps	−91.53 dBm
225 kbps	−90.25 dBm
250 kbps	−90.06 dBm

**Table 3:** Current consumptions in 41-segment transmission power.

Transmit Power (dBm)	Current (mA)	Transmit Power (dBm)	Current (mA)	Transmit Power (dBm)	Current (mA)
10.062	37.739	−1.3157	16.637	−15.688	13.005
9.3152	35.763	−2.0975	16.144	−17.207	12.791
8.2542	33.223	−2.9932	15.633	−18.484	12.612
7.1839	31.071	−4.0892	15.027	−19.539	12.509
6.8546	30.347	−4.7187	14.729	−20.784	12.395
6.2026	29.153	−5.5103	14.42	−21.604	12.356
5.5377	28.042	−6.8004	14.012	−22.212	12.299
4.3618	20.562	−7.5317	13.913	−23.3	12.246
3.6703	20.005	−8.5849	14.893	−24.073	12.173
3.0454	19.349	−9.7606	14.474	−25.125	12.116
2.2062	18.613	−11.155	14.077	−26.202	12.059
0.78139	17.556	−12.904	13.65	−27.653	12.021
−0.14598	17.583	−13.856	13.325	−28.899	11.941
−0.98536	16.942	−14.407	13.219		

**Table 4:** Node experimental results.

	Packet Error Number	PER (%)	Response (TX) Average Current Consumption	Overall Average Current Consumption
Node1	7989	1.4035	8.4740 uA	35.924 uA
Node6	213	0.0374	39.852 uA	74.281 uA
Node2	3599	0.6322	10.493 uA	38.310 uA
Node7	864	0.1518	39.852 uA	74.281 uA
Node3	5426	0.9532	11.760 uA	39.845 uA
Node8	519	0.0912	39.852 uA	74.281 uA
Node4	4798	0.8429	16.106 uA	44.991 uA
Node9	1896	0.3331	39.852 uA	74.281 uA
Node5	2283	0.4011	5.6440 uA	32.516 uA
Node10	1922	0.3376	39.852 uA	74.281 uA

**Table 5:** The battery voltage of each node at the 69th day.

Experimental Group	Node 1	Node 2	Node 3	Node 4	Node 5
Battery voltage	2.659 V	2.65 V	2.66 V	2.652 V	2.669 V
Control group	Node 6	Node 7	Node 8	Node 9	Node 10
Battery voltage	2.618 V	2.614 V	2.617 V	2.606 V	2.624 V
